# Tumor Suppressor Role of hsa-miR-193a-3p and -5p in Cutaneous Melanoma

**DOI:** 10.3390/ijms21176183

**Published:** 2020-08-27

**Authors:** Beatrice Polini, Sara Carpi, Stefano Doccini, Valentina Citi, Alma Martelli, Sara Feola, Filippo Maria Santorelli, Vincenzo Cerullo, Antonella Romanini, Paola Nieri

**Affiliations:** 1 Department of Pharmacy, University of Pisa, 56126 Pisa, Italy; beatrice.polini@farm.unipi.it (B.P.); valentina.citi@unipi.it (V.C.); alma.martelli@unipi.it (A.M.); paola.nieri@unipi.it (P.N.); 2 Molecular Medicine for Neurodegenerative and Neuromuscular Diseases Unit, IRCCS Stella Maris Foundation, 56128 Pisa, Italy; stefano.doccini@gmail.com (S.D.); filippo3364@gmail.com (F.M.S.); 3 Laboratory of ImmunoViroTherapy (IVTLab), Drug Research Program (DRP), Translation Immunology Program (TRIMM), iCAN Precision Cancer Medicine, University of Helsinki, 00014 Helsinki, Finland; sara.feola@helsinki.fi (S.F.); vincenzo.cerullo@helsinki.fi (V.C.); 4 Medical Oncology Unit, Azienda Ospedaliero-Universitaria Pisana, 56126 Pisa, Italy; amvromanini@gmail.com

**Keywords:** cutaneous melanoma, miR-193a-3p, miR-193a-5p, tumor suppressor, microRNA

## Abstract

Background: Remarkable deregulation of several microRNAs (miRNAs) is demonstrated in cutaneous melanoma. hsa-miR-193a-3p is reported to be under-expressed in tissues and in plasma of melanoma patients, but the role of both miR-193a arms in melanoma is not known yet. Methods: After observing the reduced levels of miR-193a arms in plasma exosomes of melanoma patients, the effects of hsa-miR-193a-3p and –5p transfection in cutaneous melanoma cell lines are investigated. Results: In melanoma cell lines A375, 501Mel, and MeWo, the ectopic over-expression of miR-193a arms significantly reduced cell viability as well as the expression of genes involved in proliferation (ERBB2, KRAS, PIK3R3, and MTOR) and apoptosis (MCL1 and NUSAP1). These functional features were accompanied by a significant downregulation of Akt and Erk pathways and a strong increase in the apoptotic process. Since in silico databases revealed TROY, an orphan member of the tumor necrosis receptor family, as a potential direct target of miR-193a-5p, this possibility was investigated using the luciferase assay and excluded by our results. Conclusions: Our results underline a relevant role of miR-193a, both -3p and -5p, as tumor suppressors clarifying the intracellular mechanisms involved and suggesting that their ectopic over-expression could represent a novel treatment for cutaneous melanoma patients.

## 1. Introduction

Cutaneous melanoma, a malignant tumor arising from melanocytes, is the most aggressive and fatal form of skin cancer with increasing incidence worldwide over the past several decades [1]. Targeted therapy and immunotherapy have considerably improved the outcome of advanced melanoma patients, but resistance and toxicity, as well as incomplete therapeutic response, often arise [2,3,4]. Therefore, melanoma research still needs both to develop other treatments and associations, which can prolong survival, and to identify biomarkers to early and accurately identify non-responding patients or patients who are resistant to such treatments. 

To overcome these unmet clinical needs, molecular mechanisms involved in melanoma development and progression need to be deeply investigated.

MicroRNAs (miRNAs or miRs) are short non-coding RNAs acting as master regulators of cellular processes through the simultaneous modulation of the expression levels of hundreds of mRNAs. Therefore, miRNAs are pleiotropic modulators of a wide array of biological processes, including proliferation, differentiation, apoptosis, and development, influencing cell destiny [5,6,7,8,9]. The ability of miRNAs to simultaneously silence multiple targets makes their transcriptional modulation of great interest as a novel pharmacological strategy in cancer treatment [9,10], where a single pathway of resistance leads to therapy failure [11].

Deregulation of miRNAs in cancer and cutaneous melanoma is reported in both tissues and biofluids, where several miRNAs emerged as oncogenes or tumor suppressors with key functions in carcinogenesis and cancer progression [12]. In particular, *Homo sapiens* (hsa)-miR-193a caught our attention because its role in cutaneous melanoma has only partially been explored [13,14]. MiR-193a is a member of the miR-193 family including miR-193a, miR-193b, and miR-193c. MiR-193a coding gene, named MIR193A, is located on human chromosome 17q11.2 in an active transcriptional region. During miRNA biogenesis, two mature miRNAs (arms) are generated from pre-miR-193a: miR-193a-3p (sequence: AACUGGCCUACAAAGUCCCAGU, accession number: MIMAT0000459) and miR-193a-5p (sequence: UGGGUCUUUGCGGGCGAGAUGA, accession number: MIMAT0004614). Both miR-193a arms are expressed in all normal tissues in physiological conditions [15,16] and they are reported to be deregulated in different types of human cancers, in which they can act as tumor suppressors or oncogenes. In detail, in several tumor tissues, miR-193a-3p appeared downregulated and showed tumor-suppressive properties [17,18,19,20,21,22,23,24]. Conversely, Yi and colleagues reported a significant upregulation of miR-193a-3p in esophageal squamous cell carcinoma tissues, where it acts as an oncogene [25]. Regarding melanoma, the down-expression of miR-193a-3p in melanoma tissues is already reported [13,26,27]. The miR-193a-5p expression in melanoma cells and tissues has been studied [26,27] and reports show it as downregulated in various cancer types [21,28,29,30,31,32] but also upregulated in prostate cancer tissues [33]. As far as circulating levels is concerned, circulating miR-193a-3p has been found deregulated in several types of tumors [34,35,36], while circulating miR-193a-5p has been only reported upregulated in plasma of hepatocellular carcinoma [37]. In melanoma, circulating miR-193a-3p was at lower levels in the plasma of patients at all stages of disease compared to healthy donors [14], whereas the circulating expression levels of miR-193a-5p in biofluids of melanoma patients have not been investigated yet.

Concerning their activity in cancer cells, miR-193a -3p and -5p were observed to silence several targets such as Kirsten rat sarcoma viral oncogene homolog (KRAS) [38,39], Erb-B2 receptor tyrosine kinase 2 (ERBB2) [40], phosphoinositide-3-kinase regulatory subunit 3 (PIK3R3) [21], mechanistic target of rapamycin kinase (mTOR) [21], myeloid leukemia cell differentiation (MCL1) [17], and nucleolar and spindle associated protein 1 (NUSAP1) [38]. All these targets are considered to be associated with carcinogenesis or cancer progression [41,42,43,44]. 

In a pilot study, we verified the occurrence of reduced levels of both miR-193a arms expression in exosomes plasma-derived from advanced melanoma patients and healthy subjects. The present study evaluated the tumor suppressor activity of miR-193a-3p and -5p in cultured melanoma cells from primary tumor and metastasis. 

## 2. Results

### 2.1. Down-Expression of Exosome-Derived miR-193a Arms in Plasma of Melanoma Patients

Patient characteristics of 11 stage IV melanoma patients, enrolled from January 2015 to February 2016, are reported in Table 1. The median ages of the melanoma patients and healthy volunteers were 60 and 55 years, respectively.

**Figure 1:**
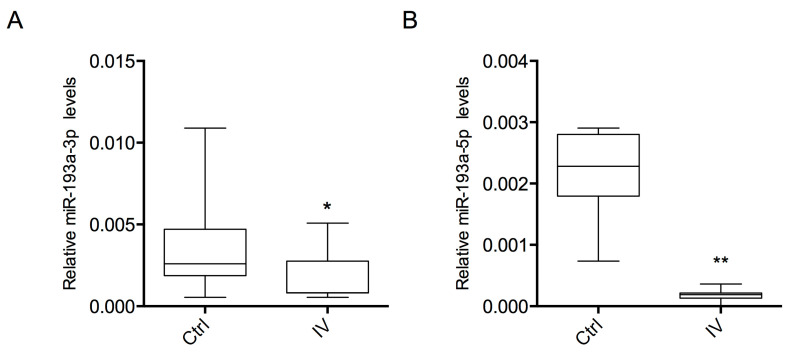
Expression levels of exosome-derived miR-193a in plasma samples. Relative expression of miR-193a-3p (**A**) and miR-193a-5p (**B**) in stage IV melanoma patients (IV) and healthy subjects (Ctrl). Statistical analysis was performed using Student’s *t*-test (* *p* < 0.05, ** *p* < 0.01).

Both miR-193a-3p and -5p showed a significant down-expression (*p* < 0.05 and *p* < 0.01, respectively) in melanoma patients compared to healthy controls (Figure 1). 

### 2.2. Ectopic Over-Expression of miR-193a Arms Inhibits Cell Viability

The ectopic over-expression of miR-193a-3p and miR-193a-5p at 10 nM in the three melanoma cell lines: A375 (ATCC^®^ Manassas, VA, USA CRL-1619, from primary cutaneous melanoma), MeWo (ATCC^®^ HTB-65, from lymph nodal cutaneous melanoma metastasis) and 501Mel (from not better specified metastatic cutaneous melanoma) caused a significant time-dependent inhibition of cell viability, compared to the correspondent scrambled miRNA transfected cells.

**Figure 2:**
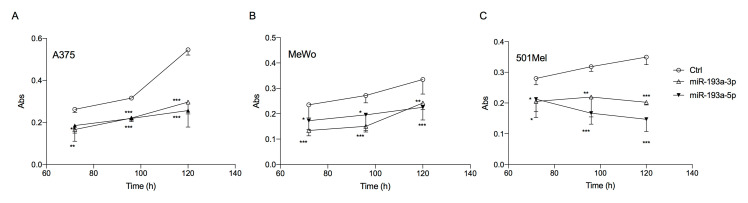
Ectopic over-expression of miR-193a arms reduces melanoma cell viability. Cell viability was analyzed by Neutral Red assay at different time points in A375 (**A**), MeWo (**B**), and 501Mel (**C**) cell lines. Data are representative of three independent experiments ± SD performed in triplicate. One-way ANOVA followed by Dunnett’s multiple comparisons test was performed; * *p* < 0.05, ** *p* < 0.01, *** *p* < 0.001 compared to the corresponding control.

Decrease in cell viability was significant after 72 h and was maintained until 120 h post-transfection in all three cell lines (Figure 2).

### 2.3. Bioinformatic miRNA Pathway Analysis and Assessment of Target Gene Downregulation

To analyze the possible biological meaning of the ectopic over-expression of miR-193a arms, all predicted target genes of miR-193a arms obtained from the TargetScan database (Tables S1 and S2 in Supplementary Materials) were imported into DAVID to determine GO biological processes (BP). 

**Figure 3:**
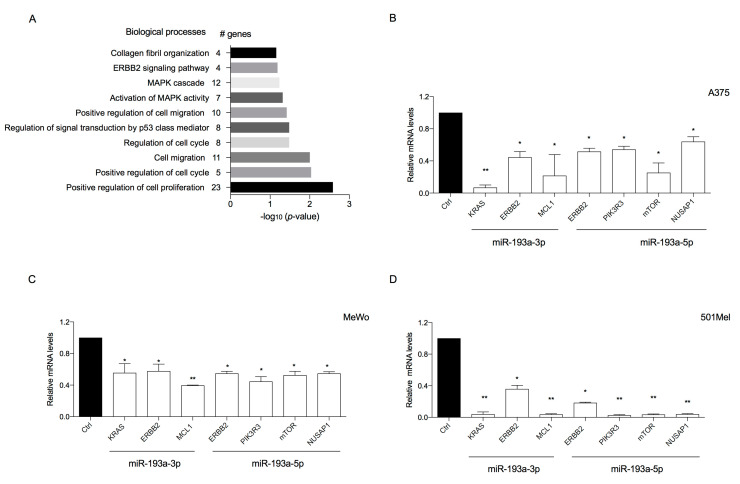
(**A**) Putative biological processes associated with target genes that can be negatively affected by ectopic over-expression of miR-193a-3p and -5p, as identified using DAVID analysis. (**B**–**D**) Ectopic over-expression of miR-193a arms reduces target genes in A375 (**B**), MeWo (**C**), and 501Mel (**D**). The expression of direct targets of miR-193a-3p (KRAS, EBBB2, and MCL1) or -5p (PIK3R3, MTOR, and NUSAP1) in melanoma cell lines following transfection with miR-193a-3p or -5p was measured by RT-qPCR. Data are normalized to β-actin and control mimic-transfected cells. Data are representative of three independent experiments ± SD performed in triplicate. Statistical analysis was performed using Student’s *t*-test (* *p* < 0.5, ** *p* < 0.01, compared to the corresponding control.)

GO analysis revealed the involvement of miR-193a arms in cancer by negatively affecting several biological processes including “mitogen-activated protein kinase (MAPK) cascade” (12 genes, *p* < 0.05), “activation of MAPK activity (7 genes, *p* < 0.05), “positive regulation of cell migration” (10 genes, *p* < 0.05), “positive regulation of cell cycle” (5 genes, *p* < 0.01), and “positive regulation of cell proliferation” (23 genes, *p* < 0.01) (Figure 3A).

To determine whether the tumor suppressor activity suggested by GO and seen with ectopic over-expression of miR-193a arms was associated with the downregulation of some of the targets previously identified, we analyzed the expression of selected mRNA transcripts in the three melanoma cell lines. 

All the chosen targets of miR-193a-3p and -5p are not only predicted by bioinformatics tools but were also validated and expressed in melanoma cells (as reported in The Human Protein Atlas). Indeed, Fan and colleagues validated KRAS as a target of miR-193a-3p [45]; Yu and colleagues validated PIK3R3 and mTOR as targets of miR-193a-5p [21]. ERBB2 is a validated target of both arms [40,46,47]; NUSAP-1 of miR-193a-5p [48] and MCL-1 of miR-193a-3p [49,50].

Over-expression of miR-193a-3p downregulated the transcriptional expression levels of KRAS, EBBB2, and MCL1. Over-expression of miR-193a-5p downregulated the transcriptional expression levels of ERBB2, PIK3R3, MTOR, and NUSAP1 (Figure 3B–D).

### 2.4. Ectopic Over-Expression of miR-193a Arms Inhibits Erk and Akt Signaling

Extracellular signal-regulated kinases (Erk) and Akt signaling are often aberrantly activated in melanoma cells, inducing cell proliferation and survival. Therefore, we analyzed the ability of miR-193a arms to modulate the protein expression level of B-Raf and the phosphorylation levels of Erk 1/2 and Akt 1/2/3.

**Figure 4:**
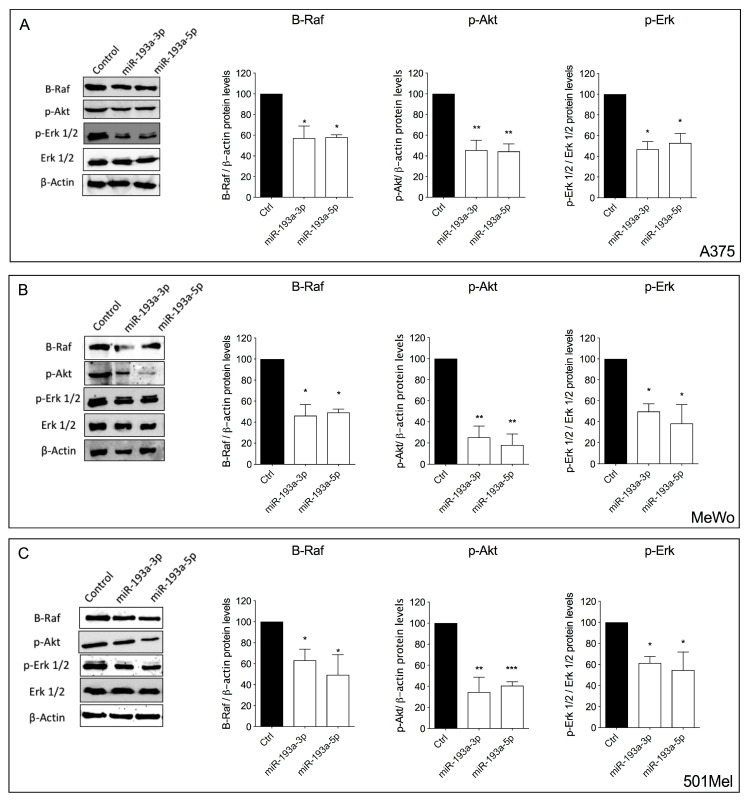
Ectopic over-expression of miR-193a arms reduces Akt and Ekt pathways activation. Protein expression levels of B-Raf, p-Akt, p-Erk 1/2, total Erk 1/2, and β-actin after ectopic over-expression of miR-193a-3p and miR-193a-5p in melanoma cells. Protein expression of B-Raf, p-Akt, p-Erk 1/2, total Erk 1/2, and β-actin in A375 (**A**), MeWo (**B**), and 501Mel (**C**) cell lines after 72 h of transfection with miRNA mimics was performed by Western blot analysis. Data are representative of three independent experiments ± SD performed in triplicate. One-way ANOVA followed by Dunnett’s multiple comparisons test was performed. * *p* < 0.05, ** *p* < 0.01, *** *p* < 0.001, compared to corresponding control.

Both miR-193a-3p and miR-193a-5p over-expression induced a significant decrease in B-Raf levels and a decrease in phosphorylation of Akt 1/2/3 and Erk 1/2 proteins in all melanoma cell lines (Figure 4). 

Noteworthily, miR-193a -3p and -5p decreased the phosphorylation of Erk 1/2 without altering the total protein levels. The observed decrease of B-Raf protein is in line with the reduced activation of Erk since B-Raf is an upstream regulator of Erk activity.

### 2.5. Ectopic Over-Expression of miR-193a Arms Induces Apoptosis

The ability of miR-193a arms to induce apoptosis in human melanoma cells was investigated by evaluating the cleavage of poly (ADP-ribose) polymerase (PARP) and the internucleosomal DNA fragmentation, phenomena that occur during early [51] and late stages of programmed cell death, respectively.

**Figure 5:**
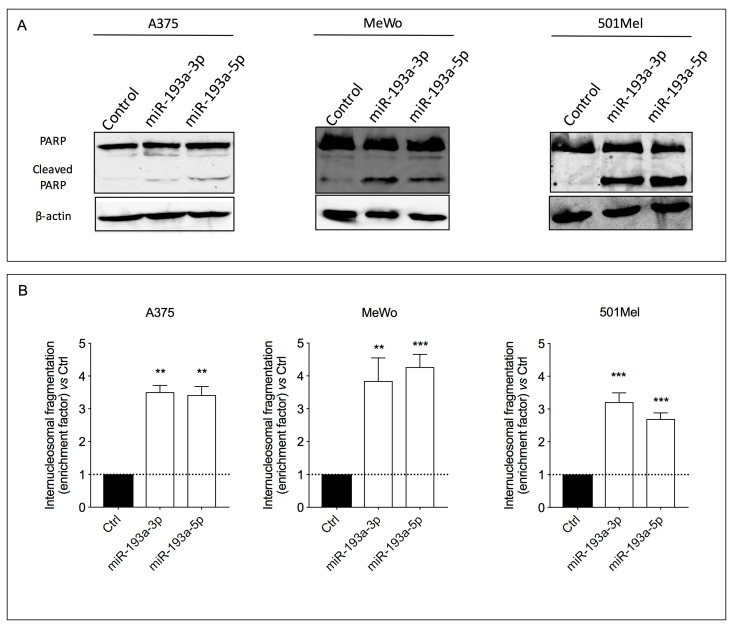
Ectopic over-expression of miR-193a arms induces apoptosis. PARP cleavage (**A**) and internucleosomal DNA fragmentation (**B**) in A375, MeWo, and 501Mel cell lines after 72 h of ectopic over-expression of miR-193a-3p and miR-193a-5p. Data are presented as means ± SD of three independent experiments performed in triplicate. Ordinary one-way ANOVA followed by Dunnett’s multiple comparisons test was performed, ** *p* < 0.01; *** *p* < 0.001 compared to the corresponding control.

The results showed that both miR-193a arms produced two bands derived from PARP cleavage (116 and 89 kDa, respectively) in the melanoma cell lysates (Figure 5A).

The pro-apoptotic effect of miR-193a was confirmed also by the internucleosomal DNA fragmentation analysis. Indeed, a significant accumulation of DNA-histone fragments (almost threefold increase) in the cytoplasmic fraction of all three cell lines compared to the respective controls was observed after miR-193a arm ectopic over-expression (Figure 5B).

### 2.6. miR-193a-5p Does Not Repress TROY Expression

To explore the molecular mechanisms involved in miR-193a-5p anti-melanoma activity, its capability to repress TROY mRNA was evaluated because TROY is a predicted (by TargetScan 7.2, Table S2) and not yet validated direct target of miR-193a-5p. 

TROY, called also TAJ or TNFRSF19 [52,53], is a gene encoding an orphan member of the tumor necrosis factor (TNF) receptor superfamily that is implicated in several cancers [54,55,56], including melanoma [57] and can promote Akt pathway [56,58]. 

**Figure 6:**
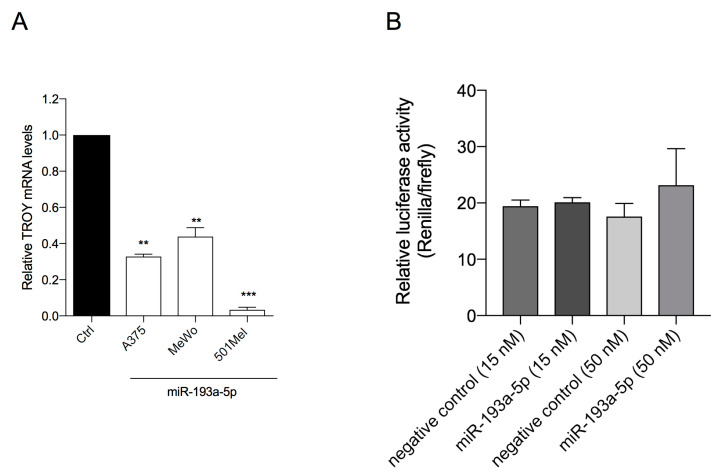
(**A**) Ectopic over-expression of miR-193a-5p reduces TROY transcriptional expression levels in A375, MeWo, and 501Mel cells. Data are representative of three independent experiments ± SD performed in triplicate. Statistical analysis was performed by Student’s *t*-test (** *p* < 0.01, *** *p* < 0.001, compared to corresponding control). (**B**) Luciferase assay analysis for miR-193a-5p and TROY mRNA 3′-UTR interaction. Luciferase assay signal did not show any downregulation by miR-193a-5p compared to the negative control.

We observed that ectopic over-expression of miR-193a-5p induced a significant decrease expression of TROY in the three cell lines (Figure 6A). To further investigate the effects miR-193a-5p, we co-transfected miR-193a-5p mimic and luciferase reporter plasmid containing wt-Luc-TROY (Figure S1). As shown in Figure 6B, the luciferase activity was not significantly repressed by miR-193a-5p over-expression, suggesting that miR-193a-5p does not directly target TROY (Figure 6B). 

## 3. Discussion

MiR-193a arms play a disease-dependent role of tumor suppressors or oncogenes, respectively associated with their under-expression in several tumor types and over-expression in others [15,16]. This trend may seem controversial, but it can be explained by the correlation between the expression of miRNAs and their target genes in different types of cells. Indeed, the biological effect of miRNA depends on the expression of each single gene target, which may present different expressions in different types of cancer cells [6,7].

For the first time, we showed that miR-193a-3p and miR-193a-5p act as tumor suppressors in melanoma cells, and both are significantly down-expressed in plasma-exosomes of melanoma patients compared to healthy controls. 

Interestingly, the arm -3p was more expressed than the -5p in both healthy subjects and melanoma patients indicating the absence of a miRNA-arm switching, i.e., the imbalance between the two miR-193a arm expression. Evidence reported that the expression of the arm of some miRNAs can change during cancer progression, accordingly with a different role played by single arm [26,59,60,61,62]. Therefore, our data showing a consistent balance of miR-193a arm expression in healthy subjects and melanoma patients are consistent with the same role of tumor suppressor here reported of miR-193a-3p and miR-193a-5p in melanoma cells. 

**Figure 7:**
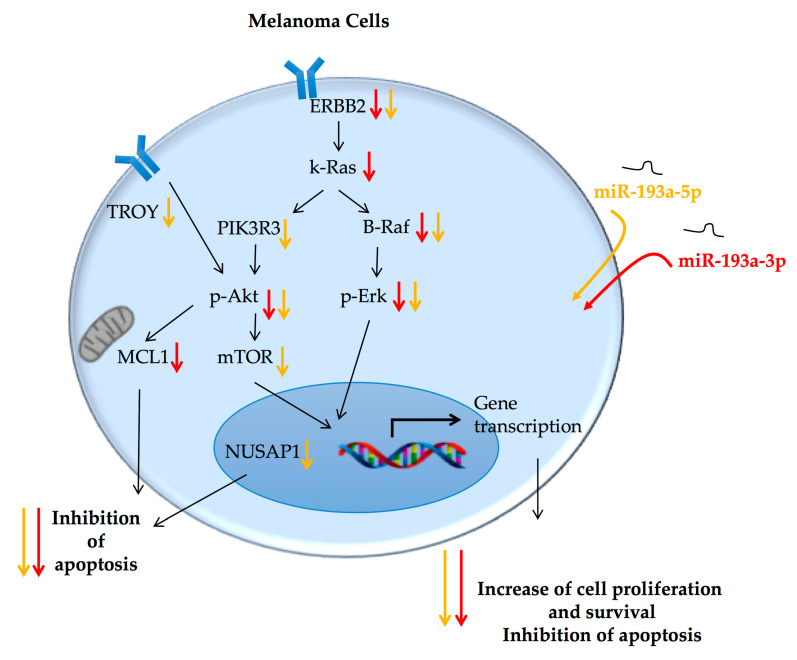
Schematic summary of the pathways involved in the tumor suppressor activity of miR-193a-3p and -5p in melanoma cells. Ectopic over-expression of miR-193a arms decrease melanoma cell proliferation and survival, and increases apoptosis, showing an interesting role of tumor suppressor. See the text for further details. The black arrows indicate progression; the orange arrows indicate a decrease of gene or protein expression after treatment with miR-193a-5p and the red arrows indicate a decrease of the gene or protein expression after treatment with miR-193a-3p.

The expression trend in exosomes from melanoma patients gave us great support in the hypothesis of a tumor suppressor role of these miRNAs in melanoma. This role was investigated on cultured melanoma cells. Independently from their primary or metastatic origin, melanoma cells decreased their viability when transfected with miRNA-193a, -3p or -5p. Bioinformatics tools and the analysis of cell expression of validated mRNA-targets for miR-193a arms revealed that the two miR-193a arms may induce a similar effect on cell viability modulating common pathways, although not targeting exactly the same genes (Figure 7).

In detail, a strong down-modulation of several cancer hallmarks strictly involved in melanoma development and progression was observed following ectopic over-expression of miR-193a, including pro-proliferative molecules and anti-apoptotic factors, previously validated as direct targets of miR-193a [41,42,63,64,65,66]. In particular, they strongly downregulated ERBB2, an epidermal growth factor receptor (EGFR), targeted by both miR-193a arms [40,46,47]. This molecule is frequently over-expressed in various cancers where it promotes an uncontrolled cellular proliferation by the activation of several effectors [67]. Among these downstream effectors, there are other molecules targeted by miR-193a-3p or miR-193a-5p: KRAS, a target of miR-193a-3p [45], and PIK3R3 and mTOR, targets of miR-193a-5p [21], which are all proteins stimulating proliferation, cell cycle progression, and cell survival in cancer cells [68,69]. Furthermore, the ectopic over-expression of each miR-193a arm induced a significant decrease of anti-apoptotic factors. In detail, the expression of MCL-1, a member of the Bcl2 family which promotes cell survival [70], was affected by miR-193a-3p [49,50], while miR-193a-5p decreased the expression of NUSAP-1 [48], a microtubule-associated protein controlling cell proliferation by governing spindle assembly, chromosome segregation, and cytokinesis, whose decrease leads to apoptosis [71].

Our results support the hypothesis that both miR-193a arms similarly modulate the same cellular processes, i.e., cell proliferation and survival, through the regulation of the expression of differential, but sometimes overlapped, specific mRNA targets.

Moreover, according to these observations, we found that both miR-193a arms significantly decreased the activation of two cell pathways often aberrantly activated in melanoma promoting cell proliferation and survival and inhibiting the apoptotic process: Erk and Akt signaling [72]. 

It is noteworthy that the ectopic over-expression of miR-193a induced a significant decrease in the phosphorylation of these proteins without affecting their total expressions. The lower activation of these pathways may be related to the ability of the miR-193a arms to directly modulate the expression of corresponding up-stream molecules, such as ERBB2 [73], KRAS [74], and PIK3R3 [75].

The ability of miR-193a-5p to directly target ERBB2 also provides a possible explanation for the observed decreased expression levels of TROY following ectopic over-expression of this arms. Indeed, a strict interaction between ErbB2 and TROY was described in glioblastoma cells [58], where Ding and colleagues reported a positive correlation of the expression levels of TROY and EGFRs, which promote each other’s activity: TROY facilitates EGFR activation and delays EGFR internalization, while EGFRs increase TROY-induced NF-κB activation. 

Altogether, our findings strongly support the tumor suppressor role of miR-193a arms and suggest their potential as therapeutic agents in melanoma through the modulation of several key pathways involved in melanoma progression. Very recently, Ylösmäki and collaborators highlighted the ability of miR-193a-3p to significantly downregulate the expression of programmed death-ligand 1 (PD-L1) in B16.OVA murine melanoma model [76], suggesting its ability to reduce melanoma immune escape. Their actions on cancer cells both by favoring apoptotic processes and reducing the expression of PD-L1 make these miRNAs particularly promising as therapeutic agents in cutaneous melanoma.

Moreover, this reported pleiotropic action of miR-193a could have the advantage of overcoming the mechanisms of resistance to target therapies due to the onset of modifications in intracellular pathways and the activation of compensatory mechanisms. For instance, the pleiotropic ability to modulate both Erk and Akt pathways, inducing a final anti-proliferative and pro-apoptotic effect, could be advantageous in melanoma treatment. Several small-molecule inhibitors of the Erk or the mTOR pathways have been developed, but clinical benefits are limited by the broad crosstalk between these two pathways and the subsequent onset of drug resistance due to activation of intracellular compensatory mechanisms following prolonged treatment [77,78]. Evidence supports the advantage of Erk/Akt dual inhibitors [78,79]. In this context, the ability of miR-193a to target molecules, which are involved in the regulation of both Erk and Akt pathways, can be promising. 

Several miRNA mimics are tested in early phase clinical trials (NCT02580552; NCT03713320; NCT03603431) [80,81], such as MesomiR 1 (ENGeneIC), a miRNA mimic that aims to replace miR-16, a tumor suppressor that is reduced in malignant pleural mesothelioma. MesomiR 1 successfully completed the phase I clinical trial and will soon start phase II [82]. Considering all the preclinical and clinical studies on miRNAs, we can speculate that in the next decade, miRNA-based drugs could improve the management of several diseases, including cancer. In this context, miR-193a may represent an interesting candidate drug against melanoma and other cancers.

## 4. Materials and Methods 

### 4.1. miRNA-193a Arms Evaluation in Plasma Exosomes

#### 4.1.1. Enrollment of Patients and Healthy Volunteers and Sample Collection 

Patient recruitment was carried out at the Department of Oncology of the University Hospital of Pisa while healthy volunteers, with similar age and sex distribution, were recruited from the Blood Donor Center of the University Hospital of Pisa. The current study was accepted by the Ethics Committee of the Great North West Area of Tuscany (395/2014 to P.N.) and it was conducted in accordance with the principles of the Declaration of Helsinki. Signed informed consent was obtained from each participant. Eleven AJCC stage IV (https://cancerstaging.org/) patients (Table 1) and eleven healthy volunteers with similar age and sex distribution entered this study. In detail, 2.5 mL of blood were drawn from each patient, collected in BD Vacutest Kima tubes containing EDTA, and centrifuged at 4 °C for 10 min at 1900× *g* for 1 h. Plasma supernatant was then aliquoted into 1.5 mL DNA LoBind tubes (Eppendorf AG, Hamburg, Germany) and stored at −80 °C until analysis. The timing of blood collection from patients was within a month after histologically proven melanoma metastasis. 

#### 4.1.2. Expression Analysis of Plasma Exosome-Derived miRNAs

The isolation of exosome-derived miRNAs from plasma samples was performed using ExoQuick-TCTM Exosome Precipitation Solution (Exiqon, Vedbaek, Denmark), according to the manufacturer’s instructions. Briefly, 250 μL of plasma were centrifuged at 3000× *g* for 15 min. Then, the supernatant was transferred to a new tube where Exoquick reagent was added in the appropriate volume. After incubation at 4 °C for 30 min, samples were centrifuged at 1500× *g* for 30 min. The obtained pellet was used for the extraction of miRNAs.

The miRNeasy Serum/Plasma Mini Kit (Qiagen, Hilden, Germany) was used for purification and extraction of miRNAs from isolated exosomes, according to the manufacturer’s instructions. The spike-in control, a *Caenorhabditis elegans* miR-39 miRNA mimic (#219610, Qiagen, Hilden, Germany), used as standard, was added in each sample during the purification, according to the manufacturer’s instructions. Samples were retro-transcribed by the miScript Reverse Transcription II Kit (Qiagen, Hilden, Germany) and the corresponding cDNA was diluted 1:3 in RNase-free water. 

The miScript SYBR-Green PCR kit (Qiagen, Hilden, Germany) was used to perform quantitative real-time PCR experiments, according to the manufacturer’s instructions, and the fluorescent signal was detected on the MiniOpticon CFX 48 real-time PCR Detection System (Bio-Rad, Hercules, CA, USA). MiScript Primer Assays specific for hsa-miR-193a-3p (MIMAT0000459) and hsa-miR-193a-5p (MIMAT0004614) were obtained from Qiagen. The miRNA expression was calculated using the Ct method and normalized to the expression of the spike-in control. Each experiment was performed in triplicate.

### 4.2. Cell Lines

Human melanoma cells A375 (ATCC^®^ CRL-1619, from primary cutaneous melanoma) and MeWo (ATCC^®^ HTB-65, from lymph node cutaneous melanoma metastasis) were obtained from the American Type Culture Collection (ATCC). 501Mel (from cutaneous melanoma metastasis) were kindly provided by Dr. Poliseno (Oncogenomics Unit, Core Research Laboratory, Istituto Toscano Tumori c/o IFC-CNR, Pisa, Italy).

All cell lines were cultured in RPMI 1640 medium (Euroclone, Milan, Italy) supplemented with 10% fetal bovine serum (FBS), 100 U/mL penicillin, and 100 μg/mL streptomycin (Euroclone, Euroclone, Milan, Italy) in a humidified atmosphere containing 5% CO_2_ at 37 °C. Cell morphology was examined under light microscopy. 

### 4.3. miRNA Mimics and Cell Transfection

miScript miRNA mimics of hsa-miR-193a-3p and hsa-miR-193a-5p were purchased from Qiagen (Hilden, Germany). The mimic scramble was used as control (Ctrl) in each experiment. Cell lines were plated at 60% of confluence and transfected with 10 nM mimic (miR-193a-3p or miR-193a-5p) or Ctrl using Lipofectamine 2000^®^ (Invitrogen, Thermo Fisher Scientific, Waltham, MA, USA), according to the manufacturer’s protocol. 

### 4.4. Cell Proliferation Assay

Cell proliferation was measured using the Neutral Red Assay (Sigma-Aldrich, Milan, Italy) as previously reported [83]. Briefly, after 24 h from seeding melanoma cells (5 × 103 cells/well) onto 96 well plates, cells were transfected with 10 nM mimic or the control (Ctrl). After 72, 96, or 120 h, 10 μL of a neutral red solution (1% acetic acid and 50% ethanol) were added to each well, and cells were incubated for 2 h at 37 °C. Optical density at 540 nm was measured using Infinite^®^ M200 NanoQuant instrument (Tecan, Salzburg, Austria).

### 4.5. Pathway Analysis

The predicted targets of human miR-193a-3p and -5p were obtained from the TargetScan database (http://www.targetscan.org/faqs.Release_7.html). The predicted targets were pooled and uploaded to Database for Annotation, Visualization, and Integrated Discovery (DAVID) (https://david.ncifcrf.gov/) [84] to Gene Ontology (GO) analysis [85]. 

### 4.6. mRNA Expression Analyses

Total RNA from cells was extracted using the RNeasy Mini Kit and, then reverse transcribed using the QuantiTect Reverse Transcription kit (Qiagen, Valencia, California, USA), according to the manufacturer’s instructions. Real-time PCR was performed using SsoFast Eva Green Supermix (Ref. 172–5201; Bio-Rad, Hercules, California, USA). The sequences of forward and reverse primers were the following: ERBB2 (F) CCTCTGACGTCCATCATCTC and (R) ATCTTCTGCTGCCGTCGCTT; K-RAS (F) CAGTAGACACAAAACAGGCTCAG and (R) TGTCGGATCTCCCTCACCAATG; TROY (F) TGCTTGCCAGGATTTTATAGGAA and (R) GACGCGATCTTCACGAGGTT; PIK3R3 (F) CTTGCTCTGTGGTGGCCGAT and (R) GACGTTGAGGGAGTCGTTGT; MTOR (F) ATGCAGCTGTCCTGGTTCTC and (R) AATCAGACAGGCACGAAGGG; MCL-1 (F) CCAAGAAAGCTGCATCGAACCAT and (R) CAGCACATTCCTGATGCCACCT; NUSAP1 (F) AGCCCATCAATAAGGGAGGG and (R) ACCTGACACCCGTTTTAGCTG; β-actin (F) 5-AACTGGACGGTAGAAGGTGAC and (R) 5-GACTTCCTGTAACAACGCATC. The mRNA expression was calculated using the ΔΔCt method. 

### 4.7. Western Blot Analysis

The analysis of protein expression by Western blotting was performed as previously described [86]. The primary antibodies used and their dilutions were the followings: anti-p-Akt1-2-3 (sc-7985-R, Santa Cruz Biotechnology), anti-ERK1 (p44)/ERK2 (p42) (Ref. sc-514302, Total ERK, Santa Cruz Biotechnology), anti-Raf-B (Ref. sc-5284 Santa Cruz Biotechnology) at 1:200 dilution; anti-phosphotyrosine204-ERK1 (p44)/ERK2(p42) (Ref. sc-7383, Santa Cruz Biotechnology) at 1:500 dilution; anti-PARP) (Ref. 9542, Cell Signaling Technology) and anti-β-actin (#MAB1501, Merck-Millipore, Burlington, MA, USA), at 1:5000 dilution overnight at 4 °C. At room temperature, the membranes were incubated with horseradish peroxidase (HRP)-labeled secondary anti-rabbit (#MAB201P, Merck-Millipore, Burlington, MA, USA) or anti-mouse antibodies (A4416, Sigma-Aldrich, Milan, Italy), for 2 h. Detection of chemiluminescence signals and densitometric analysis of blots were performed using ImageQuant LAS 4000 (GE Healthcare) and ImageLab software (Bio-Rad), respectively.

### 4.8. Cell Death Detection ELISA Plus

The cytoplasmic levels of histone-associated DNA fragments (mono- and oligo-nucleosomes), a marker of the apoptotic process, were investigated using the Cell Death Detection ELISA plus (#11774425001, Sigma-Aldrich, Milan, Italy), as previously reported by Carpi and colleagues [83].

### 4.9. Luciferase Assay 

The segments of the wild-type 3′UTRs of the gene of interest (TROY) containing predicted target sites of miR-193a-5p were cloned into the pmirGLO dual-luciferase reporter (Promega, Madison, WI, USA) (Figure 6A) (wt-Luc-TROY). The pmirGLO Vector was linearized with the DraI and XbaI restriction enzymes to generate overhangs that are complementary to the annealed oligonucleotide, which was ligated by T4 DNA ligase. Sequences of primers: TROY_top_1 AAACTATAGTAAGACCCAT, TROY_bottom_1 CTAGATGGGTCTTACTATAGTTT, TROY_seq_F TTACAACCGCCAAGAAGCTG, TROY_seq-R AAAACCTCCCACATCTCCCC. Constructs were transformed using One Shot™ Stbl3™ competent cells and the purified plasmid DNA was directly transfected into Hek294T cells.

Firefly luciferase was used as the primary reporter for miRNA regulation of the 3′UTR. Renilla luciferase was used as an internal control for normalization. HEK 293T cells were maintained in 96-well plates and co-transfected with luciferase reporters (1.2 μg/well) and oligo (15 or 50 nm of miR-193a-5p mimic or negative control). The first luciferase signal reports specific experimental conditions, and the second signal reflects transfection efficiency and cell viability. 

Luciferase activities were measured after 72 h using the Dual-Glo Luciferase Assay System (Promega) following the manufacturer’s protocol. Firefly luciferase activity was normalized to sea pansy luciferase activity.

### 4.10. Statistical Analysis

Graphic representation and statistical analysis of data were performed using GraphPad Prism 7.0 (GraphPad Software, San Diego, CA, USA). Student’s *t*-test and one-way ANOVA with Dunnett’s multiple comparison test were used for comparison of two or more than two groups, respectively. Data are presented as mean ± standard deviation (SD) of three independent experiments performed in triplicate. 

## Figures and Tables

**Table 1:** Characteristics of the study population.

Variable	%
Sex	
Male	72.7
Female	27.3
Breslow thickness	
<0.75 mm	0
>0.75 mm	100
Clark level	
I-II-III	0
IV	18.12
V	81.2
Ulceration	
Present	81.8
Absent	18.2
LDH levels	
Normal	100
Elevated	0
Patient condition	
Alive with stable disease	54.5
Alive in progression	27.3
Dead	18.2

## Supplementary Materials

The following are available online at https://www.mdpi.com/1422-0067/21/17/6183/s1.

## Abbreviations

miRNAs	microRNAs
hsa	*Homo sapiens*
KRAS	Kirsten rat sarcoma viral oncogene homolog
ERBB2	Erb-B2 receptor tyrosine kinase 2
PIK3R3	Phosphoinositide-3-kinase regulatory subunit 3
mTOR	Mammalian target of rapamycin
MCL1	Myeloid leukemia cell differentiation
NUSAP1	Nucleolar and spindle associated protein 1
FBS	Fetal bovine serum
ATCC	American Type Culture Collection
Ctrl	Control
DAVID	Database for Annotation, Visualization, and Integrated Discovery
GO	Gene Ontology
SD	Standard deviation
BP	Biological processes
MAPK	Mitogen-activated protein kinase
HRP	Horseradish peroxidase
Erk	Extracellular signal-regulated kinases
PARP	Poly ADP ribose polymerase
EGFR	Epidermal growth factor receptor
TNF	Tumor necrosis factor
PD-L1	Programmed death-ligand 1
