# Supplementary material for: Tumor Suppressor Role of hsa-miR-193a-3p and -5p in Cutaneous Melanoma
Source: Int J Mol Sci. 2020 Aug 27;21(17):6183. doi: 10.3390/ijms21176183 (PMC7503447; doi:10.3390/ijms21176183)
Supplement: Supplementary file 1 [file ijms-21-06183-s001.zip › ijms-890459 sub-table.pdf]

International Journal of
Molecular Sciences

MDPI

Supplementary Materials:

Table 1. Predicted gene targets of hsa-miR-193a-3p by TargetScanHuman version 7.2.

Target gene	Representative transcript	Gene name
SLC10A6	ENST00000273905.6	solute carrier family 10 (sodium/bile acid cotransporter), member 6
IL17RD	ENST00000296318.7	interleukin 17 receptor D
FLI1	ENST00000527786.2	Fli-1 proto-oncogene, ETS transcription factor
SLC39A5	ENST00000454355.2	solute carrier family 39 (zinc transporter), member 5
ANAPC15	ENST00000543587.1	anaphase promoting complex subunit 15
DCAF7	ENST00000310827.4	DDB1 and CUL4 associated factor 7
AP2M1	ENST00000382456.3	adaptor-related protein complex 2, mu 1 subunit
FAM84A	ENST00000295092.2	family with sequence similarity 84, member A
SRSF2	ENST00000392485.2	serine/arginine-rich splicing factor 2
FHDC1	ENST00000260008.3	FH2 domain containing 1
CBX7	ENST00000216133.5	chromobox homolog 7
SLC15A1	ENST00000376503.5	solute carrier family 15 (oligopeptide transporter), member 1
SLC16A6	ENST00000327268.4	solute carrier family 16, member 6
ERBB4	ENST00000342788.4	v-erb-b2 avian erythroblastic leukemia viral oncogene homolog 4
DOK6	ENST00000382713.5	docking protein 6
HELZ	ENST00000358691.5	helicase with zinc finger
ST6GALNAC5	ENST00000477717.1	ST6 (alpha-N-acetyl-neuraminyl-2,3-beta-galactosyl-1,3)-N-acetylgalactosaminide alpha-2,6-sialyltransferase 5
HOXD13	ENST00000392539.3	homeobox D13
MSANTD2	ENST00000239614.4	Myb/SANT-like DNA-binding domain containing 2
ZNF385B	ENST00000410066.1	zinc finger protein 385B
KRAS	ENST00000256078.4	Kirsten rat sarcoma viral oncogene homolog
ZBTB5	ENST00000307750.4	zinc finger and BTB domain containing 5
MMP19	ENST00000409200.3	matrix metallopeptidase 19
E2F6	ENST00000362009.4	E2F transcription factor 6
CCND1	ENST00000227507.2	cyclin D1
CTDSPL2	ENST00000260327.4	CTD (carboxy-terminal domain, RNA polymerase II, polypeptide A) small phosphatase like 2
OSMR	ENST00000274276.3	oncostatin M receptor
SON	ENST00000290239.6	SON DNA binding protein
STMN1	ENST00000455785.2	stathmin 1

EN2	ENST00000297375.4	engrailed homeobox 2
KLF7	ENST00000423015.1	Kruppel-like factor 7 (ubiquitous)
SMIM14	ENST00000295958.5	small integral membrane protein 14
MYCN	ENST00000281043.3	v-myc avian myelocytomatosis viral oncogene neuroblastoma derived homolog
WDR82	ENST00000296490.3	WD repeat domain 82
SLC23A2	ENST00000338244.1	solute carrier family 23 (ascorbic acid transporter), member 2
NT5DC3	ENST00000392876.3	5'-nucleotidase domain containing 3
PSEN1	ENST00000344094.3	presenilin 1
NOVA1	ENST00000465357.2	neuro-oncological ventral antigen 1
CNOT6	ENST00000393356.1	CCR4-NOT transcription complex, subunit 6
POLR2J2	ENST00000591000.1	DNA-directed RNA polymerase II subunit RPB11-b1
CLSTN1	ENST00000377298.4	calsyntenin 1
TCF4	ENST00000354452.3	transcription factor 4
UBP1	ENST00000283629.3	upstream binding protein 1 (LBP-1a)
KCNJ2	ENST00000243457.3	potassium inwardly-rectifying channel, subfamily J, member 2
SOS2	ENST00000216373.5	son of sevenless homolog 2 (Drosophila)
KCNE1	ENST00000399289.3	potassium voltage-gated channel, Isk-related family, member 1
MCL1	ENST00000369026.2	myeloid cell leukemia sequence 1 (BCL2-related)
PAK3	ENST00000372007.5	p21 protein (Cdc42/Rac)-activated kinase 3
GNAO1	ENST00000262493.6	guanine nucleotide binding protein (G protein), alpha activating activity polypeptide O
PLAG1	ENST00000316981.3	pleiomorphic adenoma gene 1
ANKRD13A	ENST00000261739.4	ankyrin repeat domain 13A
ATOH8	ENST00000306279.3	atonal homolog 8 (Drosophila)
RPS21	ENST00000370562.1	ribosomal protein S21
CALM1	ENST00000356978.4	calmodulin 1 (phosphorylase kinase, delta)
NSF	ENST00000225282.8	N-ethylmaleimide-sensitive factor
PPP2R5C	ENST00000422945.2	protein phosphatase 2, regulatory subunit B', gamma
PAK4	ENST00000599386.1	p21 protein (Cdc42/Rac)-activated kinase 4
PLAU	ENST00000372764.3	plasminogen activator, urokinase
STX16	ENST00000355957.5	syntaxin 16
ALKBH5	ENST00000399138.4	alkB, alkylation repair homolog 5 (E. coli)
AL626787.1	ENST00000595430.1	
AL590483.1	ENST00000598000.1	Uncharacterized protein; cDNA FLJ42623 fis, clone BRACE3015894

ARHGEF12	ENST0000039784 3.2	Rho guanine nucleotide exchange factor (GEF) 12
ARHGEF15	ENST0000036192 6.3	Rho guanine nucleotide exchange factor (GEF) 15
GRB7	ENST0000030918 5.3	growth factor receptor-bound protein 7
ZNF618	ENST0000028846 6.7	zinc finger protein 618
ING1	ENST0000033321 9.7	inhibitor of growth family, member 1
ING5	ENST0000031355 2.6	inhibitor of growth family, member 5
ABCB8	ENST0000035605 8.4	ATP-binding cassette, sub-family B (MDR/TAP), member 8
CNBP	ENST0000050297 6.1	CCHC-type zinc finger, nucleic acid binding protein
RUNX1T1	ENST0000052362 9.1	runt-related transcription factor 1; translocated to, 1 (cyclin D-related)
ARHGAP19	ENST0000035853 1.4	Rho GTPase activating protein 19
SIAH1	ENST0000038000 6.2	siah E3 ubiquitin protein ligase 1
PLXNC1	ENST0000025852 6.4	plexin C1
ETV1	ENST0000043047 9.1	ets variant 1
LAMC1	ENST0000025834 1.4	laminin, gamma 1 (formerly LAMB2)
SKAP2	ENST0000034531 7.2	src kinase associated phosphoprotein 2
LAMC2	ENST0000026414 4.4	laminin, gamma 2
RGS9BP	ENST0000033417 6.3	regulator of G protein signaling 9 binding protein
KIAA0825	ENST0000051320 0.3	KIAA0825
ZNF248	ENST0000039586 7.3	zinc finger protein 248
GPR27	ENST0000030441 1.2	G protein-coupled receptor 27
INO80D	ENST0000040326 3.1	INO80 complex subunit D
LAMP2	ENST0000037133 5.4	lysosomal-associated membrane protein 2
BAK1	ENST0000044299 8.2	BCL2-antagonist/killer 1
YWHAZ	ENST0000039595 7.2	tyrosine 3-monooxygenase/tryptophan 5-monooxygenase activation protein, zeta polypeptide
CALB1	ENST0000026543 1.3	calbindin 1, 28kDa
IRF2BPL	ENST0000023864 7.3	interferon regulatory factor 2 binding protein-like
KIT	ENST0000028813 5.5	v-kit Hardy-Zuckerman 4 feline sarcoma viral oncogene homolog
FAIM2	ENST0000032063 4.3	Fas apoptotic inhibitory molecule 2
FAM131B	ENST0000044373 9.2	family with sequence similarity 131, member B
ERBB2IP	ENST0000028403 7.5	erbb2 interacting protein
TBX20	ENST0000040893 1.3	T-box 20
TMEM30A	ENST0000023046 1.6	transmembrane protein 30A
FOCAD	ENST0000038024 9.1	focadhesin

PPIF	ENST0000039457 9.3	peptidylprolyl isomerase F
SEPN1	ENST0000036154 7.2	selenoprotein N, 1
GLIPR2	ENST0000039661 3.3	GLI pathogenesis-related 2
ATP5F1	ENST0000036972 2.3	ATP synthase, H+ transporting, mitochondrial Fo complex, subunit B1
MOCS2	ENST0000045085 2.3	molybdenum cofactor synthesis 2
CXXC4	ENST0000039476 7.2	CXXC finger protein 4
ADAMTSL3	ENST0000028674 4.5	ADAMTS-like 3
EBAG9	ENST0000033757 3.5	estrogen receptor binding site associated, antigen, 9
HEG1	ENST0000031112 7.4	heart development protein with EGF-like domains 1
BCL2L2	ENST0000025040 5.5	BCL2-like 2
MYLK	ENST0000036077 2.3	myosin light chain kinase
TGFBR3	ENST0000021235 5.4	transforming growth factor, beta receptor III
FAT4	ENST0000039432 9.3	FAT atypical cadherin 4
SNX27	ENST0000036884 3.3	sorting nexin family member 27
ARMC1	ENST0000027656 9.3	armadillo repeat containing 1
IGFBP5	ENST0000023381 3.4	insulin-like growth factor binding protein 5
SORCS1	ENST0000036969 8.1	sortilin-related VPS10 domain containing receptor 1
GDF11	ENST0000025786 8.5	growth differentiation factor 11
DYNLL2	ENST0000057999 1.2	dynein, light chain, LC8-type 2
NAV3	ENST0000022832 7.6	neuron navigator 3
ZC3H11A	ENST0000033212 7.4	zinc finger CCCH-type containing 11A
SRSF6	ENST0000024402 0.3	serine/arginine-rich splicing factor 6
KDM5C	ENST0000037540 1.3	lysine (K)-specific demethylase 5C
GREM1	ENST0000056067 7.1	gremlin 1, DAN family BMP antagonist
TBL1XR1	ENST0000043006 9.1	transducin (beta)-like 1 X-linked receptor 1
TNFRSF21	ENST0000029686 1.2	tumor necrosis factor receptor superfamily, member 21
ETS1	ENST0000053161 1.1	v-ets avian erythroblastosis virus E26 oncogene homolog 1
GSG1L	ENST0000038089 8.2	GSG1-like
AJUBA	ENST0000026271 3.2	ajuba LIM protein
LRRC8A	ENST0000037260 0.4	leucine rich repeat containing 8 family, member A
MAP3K3	ENST0000036135 7.3	mitogen-activated protein kinase kinase kinase 3
NT5E	ENST0000036965 1.3	5'-nucleotidase, ecto (CD73)
TCEA2	ENST0000039505 3.3	transcription elongation factor A (SII), 2

CAPRIN1	ENST0000034139 4.4	cell cycle associated protein 1
PPTC7	ENST0000035430 0.3	PTC7 protein phosphatase homolog (S. cerevisiae)
HYI	ENST0000037243 2.1	hydroxypyruvate isomerase (putative)
C18orf32	ENST0000057982 0.1	chromosome 18 open reading frame 32
TANC2	ENST0000042478 9.2	tetratricopeptide repeat, ankyrin repeat and coiled-coil containing 2
SPOPL	ENST0000028009 8.4	speckle-type POZ protein-like
SCYL3	ENST0000036777 1.6	SCY1-like 3 (S. cerevisiae)
RGL1	ENST0000030468 5.4	ral guanine nucleotide dissociation stimulator-like 1
XXbac-BPG32J3.20	ENST0000046128 7.1	
ANKFY1	ENST0000034165 7.4	ankyrin repeat and FYVE domain containing 1
TAOK1	ENST0000026171 6.3	TAO kinase 1
IGSF3	ENST0000036948 6.3	immunoglobulin superfamily, member 3
SLC30A9	ENST0000026445 1.7	solute carrier family 30 (zinc transporter), member 9
CTIF	ENST0000025641 3.3	CBP80/20-dependent translation initiation factor
KLHL2	ENST0000053812 7.1	kelch-like family member 2
SPECC1L	ENST0000031432 8.9	sperm antigen with calponin homology and coiled-coil domains 1-like
FAM168B	ENST0000040918 5.1	family with sequence similarity 168, member B
PTEN	ENST0000037195 3.3	phosphatase and tensin homolog
JMY	ENST0000039613 7.4	junction mediating and regulatory protein, p53 cofactor
TMEM260	ENST0000026155 6.6	transmembrane protein 260
RAD51L3-RFFL	ENST0000059303 9.1	Uncharacterized protein
MMP16	ENST0000028661 4.6	matrix metallopeptidase 16 (membrane-inserted)
GPR146	ENST0000029746 8.3	G protein-coupled receptor 146
NRIP1	ENST0000040019 9.1	nuclear receptor interacting protein 1
BAZ2A	ENST0000037944 1.3	bromodomain adjacent to zinc finger domain, 2A
TP53RK	ENST0000037210 2.3	TP53 regulating kinase
TPM2	ENST0000037830 0.5	tropomyosin 2 (beta)
SF3B1	ENST0000033550 8.6	splicing factor 3b, subunit 1, 155kDa
PRR14L	ENST0000043448 5.1	proline rich 14-like
ABI2	ENST0000029585 1.5	abl-interactor 2
AC010336.1	ENST0000053927 8.1	Uncharacterized protein
RFFL	ENST0000031524 9.7	ring finger and FYVE-like domain containing E3 ubiquitin protein ligase
ADCY9	ENST0000029401 6.3	adenylate cyclase 9

FAM178A	ENST00000238961.4	family with sequence similarity 178, member A
SP8	ENST00000418710.2	Sp8 transcription factor
SYT1	ENST00000457153.2	synaptotagmin I
ATXN1	ENST00000244769.4	ataxin 1
DBN1	ENST00000512501.1	drebrin 1
LRP4	ENST00000378623.1	low density lipoprotein receptor-related protein 4
TESK2	ENST00000372084.1	testis-specific kinase 2
RSF1	ENST00000308488.6	remodeling and spacing factor 1
TGFB2	ENST00000366930.4	transforming growth factor, beta 2
MAPK10	ENST00000395169.3	mitogen-activated protein kinase 10
MED14	ENST00000324817.1	mediator complex subunit 14
PCDH1	ENST00000503492.1	protocadherin 1
GSR	ENST00000221130.5	glutathione reductase
CD34	ENST00000356522.4	CD34 molecule
DNAJC13	ENST00000260818.6	DnaJ (Hsp40) homolog, subfamily C, member 13
GDPD5	ENST00000443276.2	glycerophosphodiester phosphodiesterase domain containing 5
CLOCK	ENST00000309964.4	clock circadian regulator
ZFYVE26	ENST00000347230.4	zinc finger, FYVE domain containing 26
PPARGC1A	ENST00000264867.2	peroxisome proliferator-activated receptor gamma, coactivator 1 alpha
AAK1	ENST00000409085.4	AP2 associated kinase 1
INO80	ENST00000361937.3	INO80 complex subunit
SOX5	ENST00000546136.1	SRY (sex determining region Y)-box 5
ST6GALNAC3	ENST00000328299.3	ST6 (alpha-N-acetyl-neuraminyl-2,3-beta-galactosyl-1,3)-N-acetylgalactosaminide alpha-2,6-sialyltransferase 3
KMT2E	ENST00000334877.4	lysine (K)-specific methyltransferase 2E
PET117	ENST00000432901.3	PET117 homolog (S. cerevisiae)
DENND5B	ENST00000389082.5	DENN/MADD domain containing 5B
MRPL43	ENST00000477279.1	mitochondrial ribosomal protein L43
DNAJC16	ENST00000375847.3	DnaJ (Hsp40) homolog, subfamily C, member 16
DAAM2	ENST00000538976.1	dishevelled associated activator of morphogenesis 2
PTBP1	ENST00000350092.4	polypyrimidine tract binding protein 1
FAM83F	ENST00000333407.6	family with sequence similarity 83, member F
ZNF608	ENST00000504926.1	zinc finger protein 608
LUC7L3	ENST00000505658.1	LUC7-like 3 (S. cerevisiae)

RNF222	ENST00000344001.3	ring finger protein 222
DNAJB9	ENST00000249356.3	DnaJ (Hsp40) homolog, subfamily B, member 9
GXYLT1	ENST00000398675.3	glucoside xylosyltransferase 1
NOP9	ENST00000396802.3	NOP9 nucleolar protein
DDR1	ENST00000446312.1	discoidin domain receptor tyrosine kinase 1
DUSP7	ENST00000495880.1	dual specificity phosphatase 7
C6orf195	ENST00000296847.3	chromosome 6 open reading frame 195
DIO2	ENST00000438257.4	deiodinase, iodothyronine, type II
BRWD3	ENST00000373275.4	bromodomain and WD repeat domain containing 3
ARHGAP39	ENST00000377307.2	Rho GTPase activating protein 39
ZHX3	ENST00000309060.3	zinc fingers and homeoboxes 3
FOXN2	ENST00000340553.3	forkhead box N2
PLEKHA7	ENST00000531066.1	pleckstrin homology domain containing, family A member 7
CECR2	ENST00000400573.5	cat eye syndrome chromosome region, candidate 2
TRIB2	ENST00000155926.4	tribbles pseudokinase 2
NPAS4	ENST00000311034.2	neuronal PAS domain protein 4
TSC1	ENST00000298552.3	tuberous sclerosis 1
CSRNP3	ENST00000314499.7	cysteine-serine-rich nuclear protein 3
CREBRF	ENST00000540014.1	CREB3 regulatory factor
KIAA1549L	ENST00000321505.4	KIAA1549-like
PITX2	ENST00000394595.3	paired-like homeodomain 2
EED	ENST00000327320.4	embryonic ectoderm development
ENTPD1	ENST00000371207.3	ectonucleoside triphosphate diphosphohydrolase 1
LOXL4	ENST00000260702.3	lysyl oxidase-like 4
PSD4	ENST00000441564.3	pleckstrin and Sec7 domain containing 4
BRPF1	ENST00000383829.2	bromodomain and PHD finger containing, 1
RBM8A	ENST00000330165.8	RNA binding motif protein 8A
ABHD17A	ENST00000590661.1	abhydrolase domain containing 17A
CDK17	ENST00000543119.2	cyclin-dependent kinase 17
ANK3	ENST00000280772.2	ankyrin 3, node of Ranvier (ankyrin G)
MSI2	ENST00000284073.2	musashi RNA-binding protein 2
HNRNPUL2	ENST00000301785.5	heterogeneous nuclear ribonucleoprotein U-like 2
B4GALNT3	ENST00000266383.5	beta-1,4-N-acetyl-galactosaminyl transferase 3

CSRNP1	ENST00000273153.5	cysteine-serine-rich nuclear protein 1
SYN1	ENST00000340666.4	synapsin I
C6orf132	ENST00000341865.4	chromosome 6 open reading frame 132
MBNL3	ENST00000370839.3	muscleblind-like splicing regulator 3
FAF1	ENST00000396153.2	Fas (TNFRSF6) associated factor 1
RORA	ENST00000335670.6	RAR-related orphan receptor A
RBMS1	ENST00000348849.3	RNA binding motif, single stranded interacting protein 1
CLCN5	ENST00000376088.3	chloride channel, voltage-sensitive 5
DNAJB1	ENST00000254322.2	DnaJ (Hsp40) homolog, subfamily B, member 1
GSE1	ENST00000253458.7	Gse1 coiled-coil protein
PHF15	ENST00000402835.1	PHD finger protein 15
IDE	ENST00000265986.6	insulin-degrading enzyme
SNX22	ENST00000325881.4	sorting nexin 22
C2orf49	ENST00000258457.2	chromosome 2 open reading frame 49
TP53INP1	ENST00000448464.2	tumor protein p53 inducible nuclear protein 1
FLRT3	ENST00000378053.3	fibronectin leucine rich transmembrane protein 3
LGR4	ENST00000379214.4	leucine-rich repeat containing G protein-coupled receptor 4
ZNF207	ENST00000394670.4	zinc finger protein 207
DYRK1A	ENST00000339659.4	dual-specificity tyrosine-(Y)-phosphorylation regulated kinase 1A
CELSR2	ENST00000271332.3	cadherin, EGF LAG seven-pass G-type receptor 2
BICD2	ENST00000356884.6	bicaudal D homolog 2 (Drosophila)
FUBP1	ENST00000370767.1	far upstream element (FUSE) binding protein 1
FAM179A	ENST00000379558.4	family with sequence similarity 179, member A
ARNT	ENST00000358595.5	aryl hydrocarbon receptor nuclear translocator
GMFB	ENST00000554908.1	glia maturation factor, beta
NUFIP2	ENST00000225388.4	nuclear fragile X mental retardation protein interacting protein 2
KLHL15	ENST00000328046.8	kelch-like family member 15
IKZF3	ENST00000346872.3	IKAROS family zinc finger 3 (Aiolos)
FARP1	ENST00000595437.1	FERM, RhoGEF (ARHGEF) and pleckstrin domain protein 1 (chondrocyte-derived)
C1GALT1	ENST00000223122.3	core 1 synthase, glycoprotein-N-acetylgalactosamine 3-beta-galactosyltransferase, 1
SNRPD3	ENST00000215829.3	small nuclear ribonucleoprotein D3 polypeptide 18kDa
CD47	ENST00000361309.5	CD47 molecule
ACAP3	ENST00000354700.5	ArfGAP with coiled-coil, ankyrin repeat and PH domains 3

N4BP3	ENST0000027460 5.5	NEDD4 binding protein 3
SLC35D1	ENST0000023534 5.5	solute carrier family 35 (UDP-GlcA/UDP-GalNAc transporter), member D1
DST	ENST0000031243 1.6	dystonin
GABRQ	ENST0000037030 6.2	gamma-aminobutyric acid (GABA) A receptor, theta
KMT2A	ENST0000053435 8.1	lysine (K)-specific methyltransferase 2A
PDE10A	ENST0000036688 2.1	phosphodiesterase 10A
RFX3	ENST0000038200 4.3	regulatory factor X, 3 (influences HLA class II expression)
LPAR3	ENST0000044088 6.1	lysophosphatidic acid receptor 3
SLC31A1	ENST0000037421 2.4	solute carrier family 31 (copper transporter), member 1
DYRK2	ENST0000034409 6.3	dual-specificity tyrosine-(Y)-phosphorylation regulated kinase 2
PTPRF	ENST0000037241 4.3	protein tyrosine phosphatase, receptor type, F
ETV6	ENST0000039637 3.4	ets variant 6
HIC2	ENST0000040746 4.2	hypermethylated in cancer 2
TSPAN31	ENST0000054799 2.1	tetraspanin 31
KHNYN	ENST0000025134 3.5	KH and NYN domain containing
NEDD4L	ENST0000025683 2.7	neural precursor cell expressed, developmentally down-regulated 4-like, E3 ubiquitin protein ligase
BEST1	ENST0000044913 1.2	bestrophin 1
CCAR1	ENST0000026587 2.6	cell division cycle and apoptosis regulator 1
UBD	ENST0000037705 0.4	ubiquitin D
CNNM2	ENST0000036987 8.4	cyclin M2
SKP1	ENST0000035341 1.6	S-phase kinase-associated protein 1
SRSF7	ENST0000031311 7.6	serine/arginine-rich splicing factor 7
SEC31B	ENST0000037034 5.3	SEC31 homolog B (S. cerevisiae)
CADM1	ENST0000045272 2.3	cell adhesion molecule 1
CTB-54O9.9	ENST0000058601 2.1	Uncharacterized protein
MRPL3	ENST0000026499 5.3	mitochondrial ribosomal protein L3

Table 2. Predicted gene targets of hsa-miR-193a-5p by TargetScanHuman version 7.2.

Target gene	Representative transcript	Gene name
PCDHA7	ENST00000525929.1	protocadherin alpha 7
PCDHA5	ENST00000529859.1	protocadherin alpha 5
PCDHA12	ENST00000398631.2	protocadherin alpha 12
PCDHA2	ENST00000526136.1	protocadherin alpha 2
PCDHA6	ENST00000529310.1	protocadherin alpha 6
PCDHA4	ENST00000530339.1	protocadherin alpha 4
PCDHA10	ENST00000307360.5	protocadherin alpha 10
PCDHA9	ENST00000532602.1	protocadherin alpha 9
PCDHA13	ENST00000289272.2	protocadherin alpha 13
PCDHA11	ENST00000398640.2	protocadherin alpha 11
PCDHA3	ENST00000522353.2	protocadherin alpha 3
CA7	ENST00000394069.3	carbonic anhydrase VII
PCDHA1	ENST00000504120.2	protocadherin alpha 1
PCDHA8	ENST00000531613.1	protocadherin alpha 8
PCDHAC1	ENST00000253807.2	protocadherin alpha subfamily C, 1
PCDHAC2	ENST00000289269.5	protocadherin alpha subfamily C, 2
SPRED3	ENST00000587013.1	sprouty-related, EVH1 domain containing 3
GANAB	ENST00000346178.4	glucosidase, alpha; neutral AB
RPS2	ENST00000529806.1	ribosomal protein S2
C10orf62	ENST00000370640.3	chromosome 10 open reading frame 62
NLN	ENST00000380985.5	neurolysin (metallopeptidase M3 family)
DSCAML1	ENST00000527706.1	Down syndrome cell adhesion molecule like 1
UBE2D2	ENST00000398733.3	ubiquitin-conjugating enzyme E2D 2
SLC30A5	ENST00000396591.3	solute carrier family 30 (zinc transporter), member 5
BCL7C	ENST00000215115.4	B-cell CLL/lymphoma 7C
ACVR1	ENST00000263640.3	activin A receptor, type I
C15orf32	ENST00000556865.1	chromosome 15 open reading frame 32
MTRNR2L11	ENST00000604646.1	MT-RNR2-like 11 (pseudogene)
CEP57	ENST00000325542.5	centrosomal protein 57kDa
TMEM234	ENST00000373593.1	transmembrane protein 234
MTRNR2L8	ENST00000536684.1	MT-RNR2-like 8

MTRNR2L10	ENST00000545075.1	MT-RNR2-like 10
POLDIP2	ENST00000540200.1	polymerase (DNA-directed), delta interacting protein 2
MTRNR2L1	ENST00000540040.1	MT-RNR2-like 1
CDK8	ENST00000536792.1	cyclin-dependent kinase 8
FTCD	ENST00000355384.2	formimidoyltransferase cyclodeaminase
CYGB	ENST00000293230.5	cytoglobin
SFTA3	ENST00000518529.2	surfactant associated 3
USP6	ENST00000332776.4	ubiquitin specific peptidase 6 (Tre-2 oncogene)
BTG1	ENST00000256015.3	B-cell translocation gene 1, anti-proliferative
CRYBG3	ENST00000389622.2	beta-gamma crystallin domain containing 3
GOLGA6A	ENST00000290438.3	golgin A6 family, member A
ITSN1	ENST00000379960.5	intersectin 1 (SH3 domain protein)
ANKRD65	ENST00000520296.1	ankyrin repeat domain 65
LTB4R	ENST00000396789.4	leukotriene B4 receptor
IPPK	ENST00000287996.3	inositol 1,3,4,5,6-pentakisphosphate 2-kinase
GPX8	ENST00000296734.6	glutathione peroxidase 8 (putative)
TSC22D2	ENST00000361875.3	TSC22 domain family, member 2
SLC4A10	ENST00000415876.2	solute carrier family 4, sodium bicarbonate transporter, member 10
BEAN1	ENST00000299694.8	brain expressed, associated with NEDD4, 1
GOLGA8G	ENST00000329523.6	golgin A8 family, member G
OLA1	ENST00000284719.3	Obg-like ATPase 1
CTB-58E17.5	ENST00000539023.1	Uncharacterized protein; cDNA FLJ52623
METTL8	ENST00000375258.4	methyltransferase like 8
GNAQ	ENST00000286548.4	guanine nucleotide binding protein (G protein), q polypeptide
NETO2	ENST00000562435.1	neuropilin (NRP) and tolloid (TLL)-like 2
ZMYM4	ENST00000314607.6	zinc finger, MYM-type 4
WIZ	ENST00000389282.4	widely interspaced zinc finger motifs
CNTFR	ENST00000351266.4	ciliary neurotrophic factor receptor
GOLGA8F	ENST00000526619.2	golgin A8 family, member F
FBXL3	ENST00000355619.5	F-box and leucine-rich repeat protein 3
ASIC2	ENST00000225823.2	acid-sensing (proton-gated) ion channel 2
FANCD2	ENST00000383806.1	Fanconi anemia, complementation group D2
SMARCD1	ENST00000394963.4	SWI/SNF related, matrix associated, actin dependent regulator of chromatin, subfamily d, member 1

RAP2A	ENST00000245304.4	RAP2A, member of RAS oncogene family
IFFO2	ENST00000455833.2	intermediate filament family orphan 2
GABBR2	ENST00000259455.2	gamma-aminobutyric acid (GABA) B receptor, 2
GOLGA6C	ENST00000300576.5	golgin A6 family, member C
GOLGA6D	ENST00000434739.3	golgin A6 family, member D
TOM1	ENST00000382034.5	target of myb1 (chicken)
VPS13B	ENST00000395996.1	vacuolar protein sorting 13 homolog B (yeast)
ZNF687	ENST00000368879.2	zinc finger protein 687
TNFRSF19	ENST00000382263.3	tumor necrosis factor receptor superfamily, member 19
ZNF827	ENST00000379448.4	zinc finger protein 827
HDDC2	ENST00000608295.1	HD domain containing 2
MPST	ENST00000404393.1	mercaptopyruvate sulfurtransferase
XK	ENST00000378616.3	X-linked Kx blood group (McLeod syndrome)
KIAA0100	ENST00000528896.2	KIAA0100
SRSF10	ENST00000343255.5	serine/arginine-rich splicing factor 10
ANGEL1	ENST00000251089.2	angel homolog 1 (Drosophila)
RSPO4	ENST00000217260.4	R-spondin 4
POLH	ENST00000372226.1	polymerase (DNA directed), eta
METTL1	ENST00000257848.7	methyltransferase like 1
CHST14	ENST00000306243.5	carbohydrate (N-acetylgalactosamine 4-0) sulfotransferase 14
MLLT10	ENST00000377072.3	myeloid/lymphoid or mixed-lineage leukemia (trithorax homolog, Drosophila); translocated to, 10
ZNF70	ENST00000341976.3	zinc finger protein 70
DACT3	ENST00000300875.4	dishevelled-binding antagonist of beta-catenin 3
GOLGA8B	ENST00000342314.5	golgin A8 family, member B
BMF	ENST00000220446.4	Bcl2 modifying factor
MTFR1L	ENST00000474295.1	mitochondrial fission regulator 1-like
ASPRV1	ENST00000320256.4	aspartic peptidase, retroviral-like 1
DDA1	ENST00000359866.4	DET1 and DDB1 associated 1
GOLGA8A	ENST00000360553.3	golgin A8 family, member A
GOLGA6L9	ENST00000300515.8	golgin A6 family-like 9
LDB3	ENST00000542786.1	LIM domain binding 3
GDE1	ENST00000353258.3	glycerophosphodiester phosphodiesterase 1
C18orf25	ENST00000282059.6	chromosome 18 open reading frame 25

GOLGA6L9	ENST00000358583.3	Homo sapiens golgin subfamily A member 6-like protein 9-like (LOC101927658), mRNA.
C1QTNF2	ENST00000393975.3	C1q and tumor necrosis factor related protein 2
GOLGA6L10	ENST00000439287.4	Putative golgin subfamily A member 6-like protein 10
GOLGA8J	ENST00000341650.6	golgin A8 family, member J
GBA2	ENST00000378088.1	glucosidase, beta (bile acid) 2
NXF1	ENST00000531709.2	nuclear RNA export factor 1
NOVA1	ENST00000465357.2	neuro-oncological ventral antigen 1
SPOCK1	ENST00000394945.1	sparc/osteonectin, cwcv and kazal-like domains proteoglycan (testican) 1
RP13-996F3.5	ENST00000559949.1	Uncharacterized protein
GOLGA6L4	ENST00000510439.2	golgin A6 family-like 4
FRMPD4	ENST00000380682.1	FERM and PDZ domain containing 4
SRSF6	ENST00000244020.3	serine/arginine-rich splicing factor 6
CUL4A	ENST00000326335.4	cullin 4A
BCL11B	ENST00000357195.3	B-cell CLL/lymphoma 11B (zinc finger protein)
NRG1	ENST00000341377.5	neuregulin 1
RASSF2	ENST00000379400.3	Ras association (RalGDS/AF-6) domain family member 2
RPE	ENST00000429907.1	ribulose-5-phosphate-3-epimerase
GBX2	ENST00000551105.1	gastrulation brain homeobox 2
SYT12	ENST00000393946.2	synaptotagmin XII
PROX1	ENST00000366958.4	prospero homeobox 1
GOLGA8M	ENST00000563027.1	golgin A8 family, member M
GOLGA8R	ENST00000327271.10	golgin A8 family, member R
GOLGA8K	ENST00000512626.2	golgin A8 family, member K
PCDH10	ENST00000264360.5	protocadherin 10
GOLGA8H	ENST00000566740.1	golgin A8 family, member H
GOLGA8O	ENST00000509311.2	golgin A8 family, member O
CHERP	ENST00000546361.2	calcium homeostasis endoplasmic reticulum protein
COL1A1	ENST00000225964.5	collagen, type I, alpha 1
PHACTR4	ENST00000373839.3	phosphatase and actin regulator 4
HAUS5	ENST00000379045.2	HAUS augmin-like complex, subunit 5
FADS1	ENST00000350997.7	fatty acid desaturase 1
SHISA6	ENST00000441885.3	shisa family member 6
GOLGA8N	ENST00000448387.2	golgin A8 family, member N

GOLGA8I	ENST00000450802.3	golgin A8 family, member I
EEF1A1	ENST00000316292.9	eukaryotic translation elongation factor 1 alpha 1
CDK14	ENST00000380050.3	cyclin-dependent kinase 14
EBF3	ENST00000368648.3	early B-cell factor 3
RBBP6	ENST00000319715.4	retinoblastoma binding protein 6
PPP5C	ENST00000012443.4	protein phosphatase 5, catalytic subunit
EIF4EBP2	ENST00000373218.4	eukaryotic translation initiation factor 4E binding protein 2
LAMP5	ENST00000246070.2	lysosomal-associated membrane protein family, member 5
GRAMD1B	ENST00000529750.1	GRAM domain containing 1B
CCDC144A	ENST00000443444.2	coiled-coil domain containing 144A
IL1RAPL1	ENST00000378993.1	interleukin 1 receptor accessory protein-like 1
LHX1	ENST00000254457.5	LIM homeobox 1
ZNF638	ENST00000355812.3	zinc finger protein 638
BDP1	ENST00000380675.2	B double prime 1, subunit of RNA polymerase III transcription initiation factor IIIB
TMTC3	ENST00000266712.6	transmembrane and tetratricopeptide repeat containing 3
OTUD7B	ENST00000369135.4	OTU domain containing 7B
EPDR1	ENST00000423717.1	ependymin related 1
HOXA1	ENST00000355633.5	homeobox A1
ZBTB18	ENST00000358704.4	zinc finger and BTB domain containing 18
MBD6	ENST00000355673.3	methyl-CpG binding domain protein 6
HDX	ENST00000297977.5	highly divergent homeobox
SYNGR1	ENST00000328933.5	synaptogyrin 1
ELP5	ENST00000574993.1	elongator acetyltransferase complex subunit 5
PRPS1	ENST00000372435.4	phosphoribosyl pyrophosphate synthetase 1
MEX3B	ENST00000558133.1	mex-3 RNA binding family member B
SEC61A1	ENST00000243253.3	Sec61 alpha 1 subunit (S. cerevisiae)
PIP4K2B	ENST00000269554.3	phosphatidylinositol-5-phosphate 4-kinase, type II, beta
TCF15	ENST00000246080.3	transcription factor 15 (basic helix-loop-helix)
MYO9B	ENST00000595618.1	myosin IXB
KCNH8	ENST00000328405.2	potassium voltage-gated channel, subfamily H (eag-related), member 8
CLK3	ENST00000348245.3	CDC-like kinase 3
CD34	ENST00000356522.4	CD34 molecule
KMT2D	ENST00000301067.7	lysine (K)-specific methyltransferase 2D

AGO1	ENST00000373204.4	argonaute RISC catalytic component 1
IGF2	ENST00000381395.1	insulin-like growth factor 2 (somatomedin A)
MMP16	ENST00000286614.6	matrix metallopeptidase 16 (membrane-inserted)
NUAK1	ENST00000261402.2	NUAK family, SNF1-like kinase, 1
ELMOD3	ENST00000315658.7	ELMO/CED-12 domain containing 3
PSMF1	ENST00000335877.6	proteasome (prosome, macropain) inhibitor subunit 1 (PI31)
GAREM	ENST00000399218.4	GRB2 associated, regulator of MAPK1
GPR39	ENST00000329321.3	G protein-coupled receptor 39
TAOK1	ENST00000261716.3	TAO kinase 1
VCL	ENST00000372755.3	vinculin
SLC7A1	ENST00000380752.5	solute carrier family 7 (cationic amino acid transporter, y+ system), member 1
SEMA6D	ENST00000355997.3	sema domain, transmembrane domain (TM), and cytoplasmic domain, (semaphorin) 6D
MEIS1	ENST00000488550.1	Meis homeobox 1
MYO1D	ENST00000318217.5	myosin ID
HECTD3	ENST00000372168.3	HECT domain containing E3 ubiquitin protein ligase 3
NUP210	ENST00000254508.5	nucleoporin 210kDa
ANKS1A	ENST00000360359.3	ankyrin repeat and sterile alpha motif domain containing 1A
AJUBA	ENST00000262713.2	ajuba LIM protein
MTUS2	ENST00000431530.3	microtubule associated tumor suppressor candidate 2
SLC35F2	ENST00000525071.1	solute carrier family 35, member F2
FGD1	ENST00000375135.3	FYVE, RhoGEF and PH domain containing 1
SMAD9	ENST00000399275.2	SMAD family member 9
ZNF81	ENST00000338637.7	zinc finger protein 81
PAPLN	ENST00000381166.3	papilin, proteoglycan-like sulfated glycoprotein
TRAF3	ENST00000560371.1	TNF receptor-associated factor 3
CUL3	ENST00000264414.4	cullin 3
VPS4A	ENST00000254950.11	vacuolar protein sorting 4 homolog A (S. cerevisiae)
FGF14	ENST00000376131.4	fibroblast growth factor 14
GRIK3	ENST00000373091.3	glutamate receptor, ionotropic, kainate 3
TCF3	ENST00000262965.5	transcription factor 3
SENP1	ENST00000004980.5	SUMO1/sentrin specific peptidase 1
MYO5A	ENST00000399231.3	myosin VA (heavy chain 12, myoxin)
CNTN2	ENST00000331830.4	contactin 2 (axonal)

MARCH3	ENST00000308660.5	membrane-associated ring finger (C3HC4) 3, E3 ubiquitin protein ligase
ADAMTS2	ENST00000251582.7	ADAM metallopeptidase with thrombospondin type 1 motif, 2
KMT2A	ENST00000534358.1	lysine (K)-specific methyltransferase 2A
CLPB	ENST00000294053.3	ClpB caseinolytic peptidase B homolog (E. coli)
RUNX1T1	ENST00000523629.1	runt-related transcription factor 1; translocated to, 1 (cyclin D-related)
UPF1	ENST00000262803.5	UPF1 regulator of nonsense transcripts homolog (yeast)
MPRIP	ENST00000341712.4	myosin phosphatase Rho interacting protein
GATAD2B	ENST00000368655.4	GATA zinc finger domain containing 2B
RGS9BP	ENST00000334176.3	regulator of G protein signaling 9 binding protein
CSNK2A1	ENST00000349736.5	casein kinase 2, alpha 1 polypeptide
DNTTIP2	ENST00000436063.2	deoxynucleotidyltransferase, terminal, interacting protein 2
MMADHC	ENST00000303319.5	methylmalonic aciduria (cobalamin deficiency) cblD type, with homocystinuria
SLC7A6	ENST00000219343.6	solute carrier family 7 (amino acid transporter light chain, y+L system), member 6
CISD3	ENST00000439660.2	CDGSH iron sulfur domain 3
ALG6	ENST00000371108.4	ALG6, alpha-1,3-glucosyltransferase
KIAA0895L	ENST00000561621.1	KIAA0895-like
SLC22A3	ENST00000392145.1	solute carrier family 22 (organic cation transporter), member 3
LYRM4	ENST00000330636.4	LYR motif containing 4
PFAS	ENST00000314666.6	phosphoribosylformylglycinamidine synthase
EIF2B2	ENST00000266126.5	eukaryotic translation initiation factor 2B, subunit 2 beta, 39kDa
MSH5	ENST00000534153.4	mutS homolog 5
G6PC3	ENST00000269097.4	glucose 6 phosphatase, catalytic, 3
GOLGA6B	ENST00000421285.3	golgin A6 family, member B
RP13-996F3.4	ENST00000557886.1	Uncharacterized protein
KCNH1	ENST00000271751.4	potassium voltage-gated channel, subfamily H (eag-related), member 1
CNTD1	ENST00000588408.1	cyclin N-terminal domain containing 1
PBX1	ENST00000367897.1	pre-B-cell leukemia homeobox 1
