# Supplementary figures and images for: Tumor Suppressor Role of hsa-miR-193a-3p and -5p in Cutaneous Melanoma
Source: Int J Mol Sci. 2020 Aug 27;21(17):6183. doi: 10.3390/ijms21176183 (PMC7503447; doi:10.3390/ijms21176183)

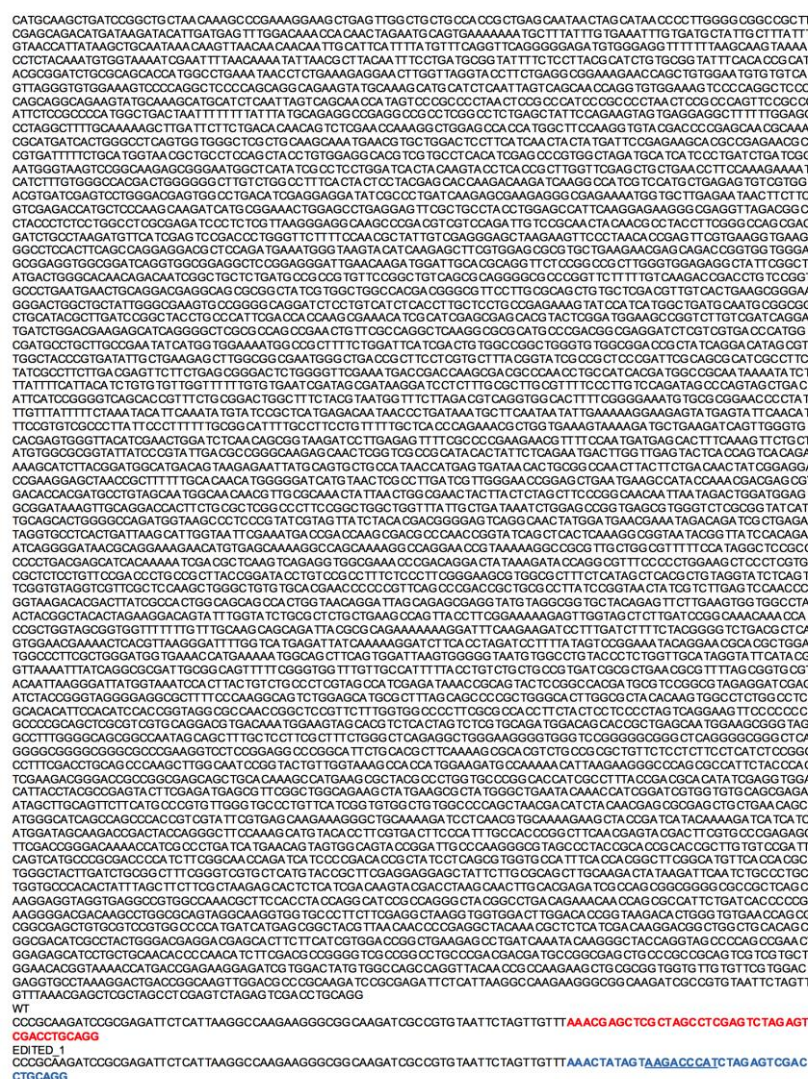

Supplement: Supplementary file 1 [file ijms-21-06183-s001.zip › ijms-890459 sub-figure.pdf]
